# Premature Ventricular Contractions at Extreme Altitude: Continuous Electrocardiographic Monitoring on Mount Everest

**DOI:** 10.1016/j.jacadv.2026.103335

**Published:** 2026-09-22

**Authors:** Domenico Angellotti, Kunjang Sherpa, Tamara Käppeli, Christoph Ryffel, Ojaswee Sherchand, Thomas Pilgrim

**Affiliations:** a Department of Cardiology, Inselspital, Bern University Hospital, University of Bern, Bern, Switzerland; b Department of Cardiology, National Academy of Medical Sciences, Bir Hospital, Kathmandu, Nepal; c Nepal Medical College and Teaching Hospital, Kathmandu, Nepal

**Keywords:** cardiac rhythm recording, extreme altitude, premature ventricular contractions

**What is the clinical question being addressed?**

Evidence on whether exposure to extreme altitude affects ventricular ectopic activity in humans is limited.

**What is the main finding?**

In healthy individuals climbing Mount Everest, ventricular ectopic activity increases modestly but progressively with altitude and is more pronounced at night.

Hypobaric hypoxia results in arterial hypoxemia in individuals exposed to extreme altitude. The hypoxemic state induces increased sympathetic activation, which may raise susceptibility to cardiac arrhythmias. In the present analysis from the SUMMIT (Cardiac Arrhythmias at Extreme Altitude) study, a prospective, high-altitude human electrophysiology study, we sought to systematically quantify premature ventricular contractions (PVCs) during ascent of Mount Everest.

The study was registered with ClinicalTrials.gov (NCT05676398) and was approved by the Nepal Health Research Council and the Cantonal Ethics Committee in Bern, Switzerland, and all participants provided written informed consent. Eligible participants underwent cardiological examination within 12 weeks before the expedition and continuous rhythm recording using a wearable continuous electrocardiogram patch (ATP-C130, ATsens). Continuous rhythm recording was repeated during the ascent from basecamp (5,300 m) to the summit of Mount Everest (8,849 m) so that every participant served as their own control. Participants with known cardiac disease were excluded per study protocol. The primary outcome was the within-subject change in PVC burden between baseline and altitude exposure. Exploratory analyses assessed changes in circadian variation in PVC burden and PVC characteristics. Rhythm recordings were independently reviewed by 2 investigators (D.A. and T.K.), blinded to altitude level. PVCs were manually identified as premature ventricular depolarizations with a widened QRS complex not preceded by a P-wave. The coupling interval was measured for each PVC and categorized as <300 ms (early PVCs) or ≥300 ms. PVCs were defined as interpolated when not followed by a compensatory pause. Artifacts were excluded during a manual review. Periods of inadequate electrocardiography signal quality were excluded from recording time. Within-patient changes in PVC burden at altitude compared to baseline were assessed using the Wilcoxon signed rank tests. Linear mixed-effects models were fitted to assess the association between altitude level and PVC burden, with altitude level included as a fixed effect and participants as the random intercept to account for repeated measurements within individuals. PVC burden was expressed as PVC/h of recording time to account for differences in recording duration between individuals. Altitude was treated as an ordinal variable, with higher values assigned to higher altitude levels (1 = basecamp [5,300 m] to camp 2 [6,500 m], 2 = camp 2 to camp 3 [7,300 m], 3 = camp 3 to camp 4 [7,900 m]/summit [8,849 m]). Given the non-normal distribution of PVC burden, a sensitivity analysis was performed using log-transformed PVC burden ([ln(PVC/h + 1)]). Rhythm recordings were stratified into daytime (06:00-18:00) and nighttime (18:00-06:00), approximately corresponding to the periods of activity and rest, respectively. A *P* value <0.05 was considered statistically significant for the primary analysis. A *P* < 0.0125 was used for exploratory analyses. Analyses were performed using R studio, version 4.1.2 (R Foundation for Statistical Computing).

Between January 25 and May 9, 2023, 41 healthy men climbing Mount Everest were prospectively recruited. Rhythm recording at baseline was completed in 35 individuals. Three participants did not reach the Everest basecamp, one did not use the patch for the climb, one lost the patch during descent, 2 patches were empty on return, and 5 had inadequate electrocardiography quality. Twenty-three individuals (median age: 32 [28-36] years) constituted the final population. The median duration of rhythm recording was 3.3 days (2.2-4.0) at baseline and 3.1 days (1.4-6.7) during the expedition at high altitude. There was no difference in duration of rhythm recording during the expedition compared to baseline (*P* = 0.45). The median peripheral oxygen saturation was 96% (95.5-97.5) at baseline and 81.5% (78-85) at basecamp (5,300 m) (Table 1). One participant climbed to 7,300 m (camp 3), 22 reached an altitude of 7,900 m (camp 4) or higher, and 10 reached the summit of Mount Everest. One participant used acetazolamide to prevent acute mountain sickness. Supplemental bottled oxygen was used during exercise by 22 participants (95.6%), starting from an altitude of 6,500 m (n = 2), reaching 7,300 m (n = 19) and 7,900 m (n = 1). One participant experienced a nonsustained ventricular tachycardia with 9 consecutive ventricular beats at a rate of 240/min during descent from camp 4 (7,900 m). None reported palpitations. Median PVC/h increased significantly during expedition compared with baseline (0.24 [0.13-0.34] vs 0.10 [0.08-0.18], *P* = 0.009). PVC/h increased by 0.43 for each increase in altitude level from basecamp to summit (β = 0.43; 95% CI: 0.18-0.68; *P* < 0.001). The association remained significant using the log-transformed PVC burden (β = 0.082; 95% CI: 0.050-0.114; *P* < 0.001). When rhythm recordings were stratified according to circadian variation, PVC/h increased significantly at extreme altitude vs baseline during nighttime (0.22 [0.08-0.50] vs 0.08 [0.03-0.12], median difference: 0.13 PVC/h, *P* < 0.001), but not during daytime (0.22 [0.0-0.71] vs 0.13 [0.05-0.25], median difference: 0.08 PVC/h, *P* = 0.063). No significant differences were observed between extreme altitude and baseline in the proportion of early PVCs (27% [1-47] vs 20% [0-33], *P* = 0.54) or interpolated PVCs (3% [1-10] vs 4% [2-20], *P* = 0.89).

**Table 1:** Baseline Characteristics and PVC Burden for Each Participant Across Altitude Levels

Participant	Age, y	Ethnic Group	HR	Number of Peaks >8,000 m Summited	SpO_2_% 1,500 m	SpO_2_% 5,300 m	PVC n Baseline <1,500 m	PVC, n at Altitude		
								During Expedition 5,300-8,849 m	Basecamp-Camp 1 5,300-6,500 m	Camp 2-Summit up to 8,849 m
Climber 1	44	European	64	1	100	90	10	6	1	5
Climber 2	29	Nepali non-Sherpa	74	6	95	84	27	1,545	511	1,034
Climber 3	21	Nepali Sherpa	52	2	96	81	7	13	0	13
Climber 4	32	Nepali Non-Sherpa	66	6	95	84	18	4	0	4
Climber 5	37	Nepali non-Sherpa	67	7	95	86	40	119	28	91
Climber 6	32	Nepali Sherpa	76	4	98	87	5	11	0	11
Climber 7	36	Nepali Sherpa	67	5	96	86	11	44	0	44
Climber 8	26	Nepali Sherpa	63	2	96	78	6	2	0	2
Climber 9	25	Nepali Sherpa	76	2	97	78	12	68	29	39
Climber 10	32	Nepali Sherpa	66	9	98	85	2	2	0	2
Climber 11	27	Nepali Sherpa	58	2	96	85	4	7	5	2
Climber 12	41	Nepali Sherpa	62	14	96	74	8	49	19	30
Climber 13	31	Nepali Sherpa	78	3	98	-	5	6	1	5
Climber 14	48	Nepali Sherpa	79	17	95	87	4	1	0	1
Climber 15	23	Nepali Sherpa	79	1	97	79	13	1	0	1
Climber 16	33	Nepali Sherpa	71	14	98	81	7	11	0	11
Climber 17	32	Nepali Sherpa	61	1	95	81	5	13	0	13
Climber 18	32	Nepali non-Sherpa	75	5	98	80	3	7	0	7
Climber 19	36	Nepali Sherpa	83	11	96	77	5	348	69	279
Climber 20	36	Nepali Sherpa	80	14	96	74	6	22	4	18
Climber 21	30	Nepali Sherpa	90	4	96	78	30	1,103	324	779
Climber 22	24	Nepali non-Sherpa	56	1	95	82	6	7	0	7
Climber 23	36	Nepali Sherpa	70	8	96	83	8	146	91	55

HR = heart rate; PVC = premature ventricular contraction; SpO₂ = peripheral oxygen saturation.

This analysis from the SUMMIT study provides novel insights into the effects of extreme altitude exposure on ventricular arrhythmic activity in healthy individuals. The main findings are the following: 1) PVC burden increased at extreme altitudes (≥5,300 m) compared with home setting conditions (<1,500 m); 2) PVC burden increased with increasing altitude up to 8,849 m; and 3) the association between altitude and ventricular ectopy was more consistent during nighttime. Previous studies have described cardiac arrhythmias and ventricular ectopy during high-altitude exposure. Prior analyses of the SUMMIT study have shown an increase in the incidence of significant bradyarrhythmias and tachyarrhythmias, as well as QT interval prolongation in healthy men exposed to extreme altitudes.^1^^,^^2^ During the 1981 American Medical Research Expedition to Mount Everest, 12 subjects reported occasional PVCs during both awake and sleep non-continuous recordings.^3^ More recently, continuous monitoring with an implantable cardiac monitor in 16 participants climbing to a maximum altitude of 7,550 m on Mount Dhaulagiri found significant bradyarrhythmias and tachyarrhythmias above 4,100 m.^4^ The present analysis extends these observations in a larger population exposed to higher altitudes by providing a longitudinal, continuous characterization of the PVC burden, with repeated measurements at different levels of oxygen pressure across altitude levels of Mount Everest. The median duration of rhythm recordings was approximately 6 days per participant. We observed a modest increase in PVC burden during extreme altitude exposure. This finding was supported by a significant association between altitude level and PVC burden. The latter was more consistent during nighttime, when the hyperventilatory response to hypoxia may be attenuated and characterized by instability, with periods of hypopnea and apnea that may exacerbate hypoxemic stress. An alternative hypothesis for the stronger association between ventricular ectopy and altitude exposure during nighttime is the rate dependence of PVCs. Many forms of ventricular ectopy occur more frequently at slower heart rates, and the nocturnal reduction in heart rate may have facilitated PVC occurrence, independent of hypoxia. Conversely, Kujaník and Mikulecky^5^ reported ventricular ectopy to occur predominantly during daytime at 1,350 m above sea level, suggesting that the circadian distribution of ventricular ectopy may differ under conditions of less-extreme hypobaric hypoxia. However, in our study, most participants used supplemental oxygen during exertion, particularly at higher altitudes, while no oxygen supplementation was used during nighttime, potentially diluting the proarrhythmic effects of hypoxia during daytime. Moreover, sleep-wake periods were not objectively assessed, the sample size was modest, and the absolute PVC burden at altitude in healthy individuals remained low. Therefore, our findings remain descriptive and do not allow mechanistic conclusions around the observed results. Our findings primarily apply to healthy male climbers, with prior altitude exposure. Whether, and to what extent, ventricular ectopy occurs in women, older individuals, non-acclimatized subjects, and patients with cardiac disease and whether it carries clinical implications in these populations remain to be established.

## EDI Statement

The study enrolled healthy adult men planning to climb Mount Everest, predominantly of Nepali ethnicity. Participant demographics are fully reported in the manuscript, and the limitations regarding generalizability are acknowledged.

## Funding support and author disclosures

The study was supported by a dedicated grant from the Swiss Polar Institute. Wearable continuous electrocardiogram patches were provided free of charge by ATsens, Seongnam, Republic of Korea. The funders had no role in the design and conduct of the study; collection, management, analysis, and interpretation of the data; preparation, review, or approval of the manuscript; and decision to submit the manuscript for publication. Dr Pilgrim has received research, travel, or educational grants for the institution without personal remuneration from Abbott, Boston Scientific, Edwards Lifesciences, and Medtronic; speaker fees and consultancy fees from Edwards Lifesciences, Boston Scientific, Medtronic, Biosensors, and Highlife; and in-kind material support from ATsens. All other authors have reported that they have no relationships relevant to the contents of this paper to disclose.
